# Supplementary material for: Sustained-input switches for transcription factors and microRNAs are central building blocks of eukaryotic gene circuits
Source: Genome Biol. 2013 Aug 23;14(8):R85. doi: 10.1186/gb-2013-14-8-r85 (PMC4054853; doi:10.1186/gb-2013-14-8-r85)
Supplement: Additional file 5 — HTML Browsable Motif Output. Zipped folder containing all WaRSwap and FANMOD motif output, viewable in a web browser. [file gb-2013-14-8-r85-S5.ZIP › HTML_browsable_motif_output/FANMOD_ath_tair9/sigs_fanmodm-2000.pvals.heatmaps.html/motif_id_38_001101002_tftype_ath_upstream_-3000_0.html]

```
BG_MODEL = FANMOD
MOTIF_ID = 38_001101002
TF_TYPE = ath
UPSTREAM = -3000_0


PVals
FN_0.2	FN_0.4	FN_0.6	FN_0.8
dg_60.genes	0.833	0.985	0.314	0.006
dg_70.genes	0.836	0.977	0.304	0.011
dg_80.genes	0.824	0.984	0.309	0.007

ZScores
FN_0.2	FN_0.4	FN_0.6	FN_0.8
dg_60.genes	-0.977	-2.125	0.488	2.119
dg_70.genes	-0.953	-2.037	0.475	2.125
dg_80.genes	-0.958	-2.117	0.512	2.161

StDevs
FN_0.2	FN_0.4	FN_0.6	FN_0.8
dg_60.genes	29840.63	37891.05	12739.78	1955.009
dg_70.genes	29478.528	38296.181	12310.265	2012.292
dg_80.genes	30253.485	38258.671	12236.558	1980.877
```
